# Role of α-Globin H Helix in the Building of Tetrameric Human Hemoglobin: Interaction with α-Hemoglobin Stabilizing Protein (AHSP) and Heme Molecule

**DOI:** 10.1371/journal.pone.0111395

**Published:** 2014-11-04

**Authors:** Elisa Domingues-Hamdi, Corinne Vasseur, Jean-Baptiste Fournier, Michael C. Marden, Henri Wajcman, Véronique Baudin-Creuza

**Affiliations:** 1 Institut National de la Santé et de la Recherche Médicale (Inserm) U779, Université Paris XI, Paris, France; 2 INSERM U955, Université Paris Est Créteil (UPEC), Hôpital Henri Mondor, Paris, France; Consejo Superior de Investigaciones Cientificas, Spain

## Abstract

Alpha-Hemoglobin Stabilizing Protein (AHSP) binds to α-hemoglobin (α-Hb) or α-globin and maintains it in a soluble state until its association with the β-Hb chain partner to form Hb tetramers. AHSP specifically recognizes the G and H helices of α-Hb. To investigate the degree of interaction of the various regions of the α-globin H helix with AHSP, this interface was studied by stepwise elimination of regions of the α-globin H helix: five truncated α-Hbs α-Hb1-138, α-Hb1-134, α-Hb1-126, α-Hb1-123, α-Hb1-117 were co-expressed with AHSP as two glutathione-S-transferase (GST) fusion proteins. SDS-PAGE and Western Blot analysis revealed that the level of expression of each truncated α-Hb was similar to that of the wild type α-Hb except the shortest protein α-Hb1-117 which displayed a decreased expression. While truncated GST-α-Hb1-138 and GST-α-Hb1-134 were normally soluble; the shorter globins GST-α-Hb1-126 and GST-α-Hb1-117 were obtained in very low quantities, and the truncated GST-α-Hb1-123 provided the least material. Absorbance and fluorescence studies of complexes showed that the truncated α-Hb1-134 and shorter forms led to modified absorption spectra together with an increased fluorescence emission. This attests that shortening the H helix leads to a lower affinity of the α-globin for the heme. Upon addition of β-Hb, the increase in fluorescence indicates the replacement of AHSP by β-Hb. The CO binding kinetics of different truncated AHSP^WT^/α-Hb complexes showed that these Hbs were not functionally normal in terms of the allosteric transition. The N-terminal part of the H helix is primordial for interaction with AHSP and C-terminal part for interaction with heme, both features being required for stability of α-globin chain.

## Introduction

The human adult hemoglobin (Hb A) is a tetrameric protein containing two α- and two β-chains, each associated with a heme molecule. It is now admitted that the α and β subunits do not associate spontaneously to form α_2_β_2_ Hb. The scheme of Hb biosynthesis is the following: firstly Alpha hemoglobin stabilizing protein (AHSP), the α-Hb chaperone, binds to the α-globin (without heme molecule) or α-Hb (with heme), maintaining it in a soluble state [1], secondly AHSP is replaced by β-hemoglobin chains (β-Hb) to form an αβ dimer, and tetramers in a further step [2], [3]. Unlike the free β-Hb which are soluble and form homologous tetramers, freshly synthesized α-Hb chains are unstable molecules. Spontaneously they form hemichromes, which precipitate and generate reactive oxygen species (ROS) within the erythrocyte precursors of the bone marrow leading to apoptosis and ineffective erythropoiesis [4]. AHSP is a 102 amino-acid protein, synthesized at a high concentration in the erythroid precursors, which forms a stable soluble heterodimer with α-Hb [1]. This α chaperone specifically recognizes the G and H helices of α-Hb, a region which also binds to the β-Hb subunit [5], [6]. *In vitro*, it has been demonstrated that AHSP inhibits ROS production by α-Hb and it was hypothesized that AHSP might protect erythroid cells from α-Hb toxicity by maintaining this subunit in a stable state prior to its incorporation into Hb A [7]. At least *in vitro*, AHSP facilitates folding of newly formed α-chains and promotes Hb A assembly [7]. More recently we have shown that redox properties and stability of α-Hb are modified by AHSP [8]. The presence of AHSP facilitates reduction of the oxidized α-Hb chain which could trigger its release from AHSP toward its final Hb β-chain partner producing functional ferrous Hb-tetramers [8]. These different data emphasize the important role of AHSP in Hb biosynthesis.

A defect in the production of one of the globin chains in the red blood cell (RBC) precursors results in various degrees of anemia and thalassemia syndromes, for example an α-thalassemia in the case of reduced α-globin production [9]. In human, the β-globin chain is encoded by a single gene localized on each of the chromosomes 11 and the α-globin chain by two genes carried by each copy of chromosome 16. Studies in mice model have shown that AHSP could play a role in Hb disorders. In mice, the loss of AHSP (AHSP^−/−^) led to reticulocytosis indicating a shortened erythrocyte half-life, abnormal erythrocyte morphology with intracellular denatured Hb inclusion and increased ROS with subsequent cellular oxidative damage as observed in thalassemias [1]. In addition, the loss of AHSP in β-thalassemic mice exacerbated thalassemic syndrome [10] and its loss in α-thalassemia silent carrier α-/αα, (3 functional α-genes) led to more severe erythroid pathologies (less hemoglobin, more reticulocytes) than in only α-thalassemia silent carrier mice.

**Figure 1:**
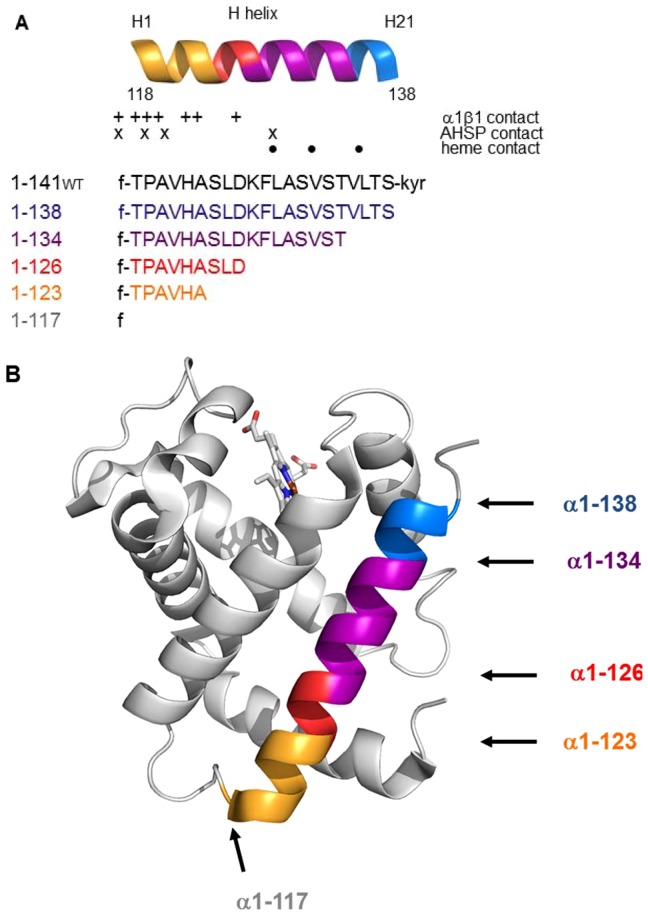
H helix of human α-globin. (A) H helix sequence of the different truncated α-globins. The symbols indicate the amino-acids interacting with β-globin within the same αβ dimer (+) and those interacting with the heme molecule (•) [14]. The (x) symbol indicates the amino-acids interacting with AHSP. (B) Three-dimensional view of α-Hb. The representation was obtained from crystallographic data of oxyHb (2HHB) using the Pymol software. The truncated regions of the H helix investigated in this work are represented in blue (α-Hb1-138), purple (α-Hb1-134), red (α-Hb1-126), orange (α-Hb1-123) and grey (α-Hb1-117). The GH corner is shown in yellow.

In human α-thalassemias, the reduced availability of α-globin chains may be due to several molecular mechanisms, the most common being gene deletion [11]. However, in the past few years, the number of α chain Hb variants discovered with point mutations, short deletions, or insertions which resulted in α-thalassemia phenotypes, has increased [11]. Variants in which the contact region between α and β-subunits is altered, were often considered as the cause of these α-thalassemia phenotypes. The discovery of AHSP allows to consider an alternate explanation of some non-deletional α-thalassemias by impairing interaction between α-Hb and AHSP (Figure 1A). In a first study, it has been reported that two elongated α-Hb variants, Hb Constant Spring and Paksé associated with an α-thalassemia phenotype led to an impaired binding to AHSP [12]. Beside the elongated chains, point mutations with a single amino acid change were reported also to cause this clinical presentation. Our group has shown that Hb Groene Hart [α119(H2)Pro→Ser], a variant common in North Africa and in Southern Italy, causes a defect in the association with AHSP and behaves as an α^+^-thalassemia with two functional α-genes [13]. In a similar way, Lacerra *et al* described Hb Foggia [α117(GH5)Phe →Ser] an α-2 gene variant that led to an α-thalassemia phenotype in the carriers [14]. The mutated mRNA α-globin of this variant was present at normal level in the reticulocytes but no abnormal chains were detected suggesting that it was synthesized but very rapidly degraded likely due to impaired interaction with AHSP [14]. Recent studies, from our group and from others, demonstrated a similar defect in AHSP binding for an increasing number of α-Hb variants [15], [16]. The region 95–137 of α-Hb, composed by G and H helices, plays a predominant role in the interaction between α-Hb and AHSP [6]. In addition, the region 103–124 allows together the binding to AHSP and to β-Hb. Some amino-acids interact with AHSP, others only with β-Hb within an α1β1 dimer [17] and three (103, 117, 119) are involved in both contacts as shown in the figure 1A.

In this work, we investigated the minimal length of the α-chain helix H allowing in the first step the formation of AHSP^WT^/α-Hb complex and in the second one that of the stable tetramer. For this purpose, we engineered wild type α-Hb1-141 with a N-terminal Gly-Pro-Leu-Gly-Ser peptide (α-Hb^WT^) and five truncated α-Hbs in which the H helix and the C-terminal were altered (Table 1). These truncated α-Hbs (α-Hb1-138, α-Hb1-134, α-Hb1-126, α-Hb1-123, α-Hb1-117) were selected from the list of α mutants already described (Table 2) [18]. They extended from the complete removal of helix H (α-Hb1-117) to the simple deletion of the three C-terminal residues located at the end of the helical structure (α-Hb1-138) (Table 1, Figure 1B). These molecules were engineered in the presence of wild type AHSP with a N-terminal Gly-Pro-Leu-Gly-Ser peptide (AHSP^WT^) using the co-expression vector that allows one to produce at high yield α-Hb as a complex with AHSP^WT^ [19]. We studied their stability during the three steps of the process: i) co-expression with AHSP^WT^ in bacteria, ii) solubilization of the products and iii) their purification. We studied the consequences of the different truncations on the interaction between the resulting α-Hbs and AHSP^WT^ or heme molecule by absorbance spectroscopy, circular dichroism (CD) and dynamic light scattering (DLS). The formation of α_2_β_2_ tetramers was investigated by fluorescence studies and by kinetics of CO recombination after flash photolysis.

**Table 1:** Different truncated α-Hbs produced in *E. coli* with AHSP.

Residue	Mutation	Name of truncated α chain	mass of GST-α-Hb (Da)*
139(H21)	Lys→Stop	α-Hb1-138	41 410
135(H17)	Val→Stop	α-Hb1-134	41 110
127(H9)	Lys→Stop	α-Hb1-126	40 270
124(H6)	Ser→Stop	α-Hb1-123	39 960
118(H1)	Thr→Stop	α-Hb 1-117	39 160

*Masses are equal to 42 602 Da and 38 665 Da for GST-α-Hb^WT^ and GST-AHSP^WT^, respectively.

**Table 2:** Natural variants in the G and H helices and C terminal extremity altering the sequence length of α-Hb.

Position	Hb Name	Affected α	Mutation	Length**
*G Helix*				
14		α2	α107 delT	132
*H helix*				
14	Fez	α1	α1-131	132
17	Senlis	α1	α134 delT	136
15	Pak or Num Po	α1	α132 insT TAA→TAT	175
*C-term*	Constant Spring	α2	α142→Gln TAA→ CAA	172
	Icaria	α2	α142→Lys TAA→AAA	172
	Koya Dora	α2	α142→Ser TAA→TGA	172
	Seal Rock	α2	α142→Glu TAA→GAA	172
	Paksé	α2	α142→Tyr TAA→TAT	172
	Natal	α2	α140→0 TAC→ TAA	139
				
	Wayne	α2 or α1	α139 delA	146

The data were collected from HbVar, the globin gene server (http://globin.cse.psu.edu/globin/hbvar/).

These different mutations led to an α-thalassemic phenotype more or less pronounced.

*the production of α-globin is under the control of two genes α1 and α2-globin genes (*HBA1* and *HBA2* respectively)

**Length expressed as number of amino-acid residues;

del, deletion; ins, insertion;

## Results

### Expression and purification of the truncated α-Hbs

**Figure 2:**
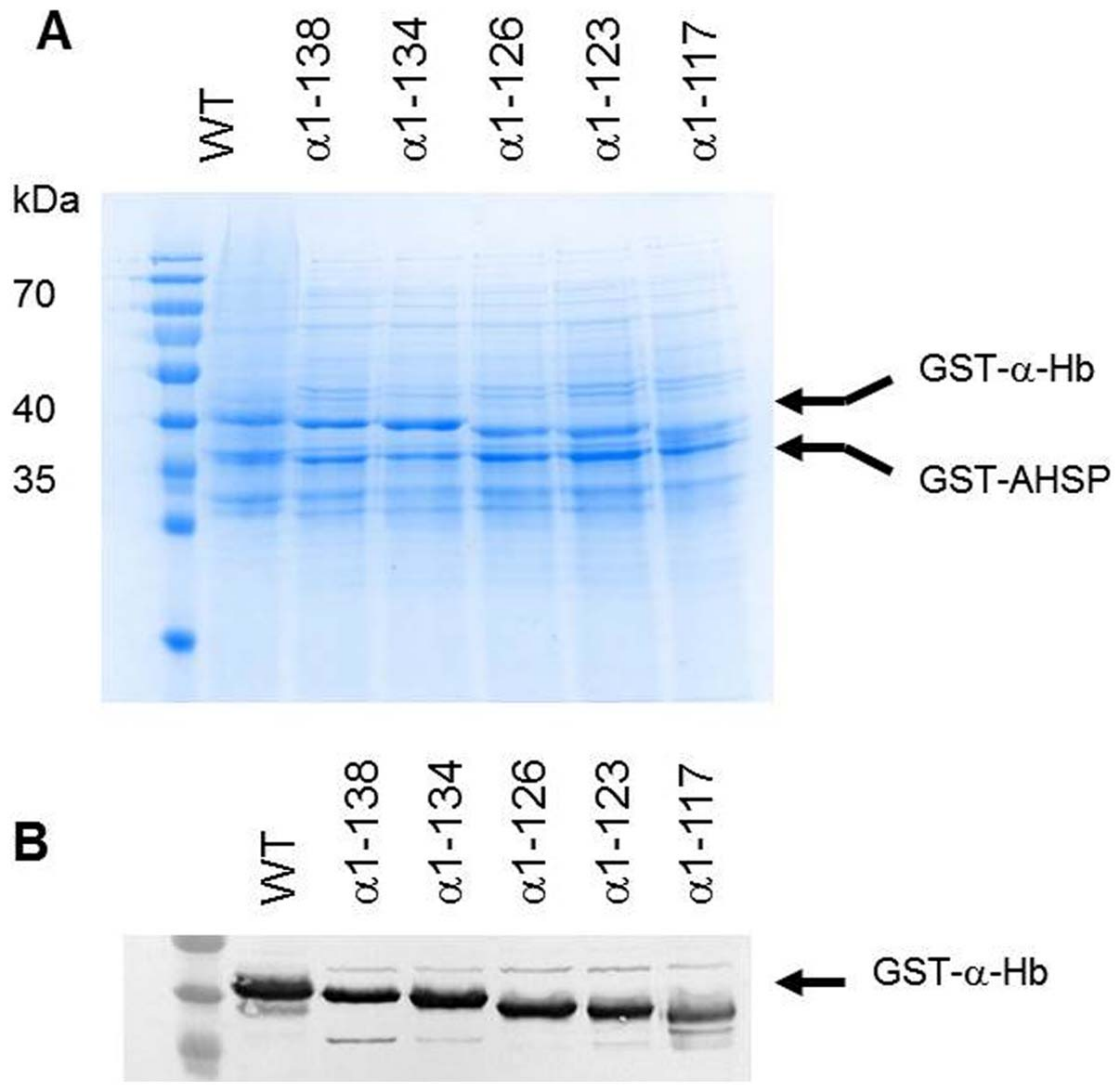
Co-expression of truncated α-Hbs and AHSP^WT^ in *E.coli* BL21(DE3) cells. After 4 h induction by 0.2 mM IPTG, the *E. coli* cells containing the different pGEX-α-AHSP were lysed and total cell lysates were subjected to SDS-PAGE (12%) analysis (A) and Western Blotting using anti-α globin antibodies (B). All samples were prepared by boiling in SDS loading buffer. In all gels, GST-α-Hb^WT^ and GST-AHSP^WT^ are indicated by arrows. PAGE ruler™ prestained protein ladder (Fermentas Thermo Fisher Scientific, Waltham, MA, USA) was analyzed in the first lane.

Five α-Hbs with shortened H helix were co-expressed with AHSP^WT^. The presence of the truncated α-Hbs was analyzed at three steps of protein production by SDS-polyacrylamide gel electrophoresis (SDS-PAGE) and Western blot analysis (Figure 2, Table 3). After 4 h induction, the whole cell lysate of the different truncated α-Hbs showed two major bands, 42 kDa and 38.6 kDa, corresponding to the GST-α-Hbs and GST-AHSP^WT^ proteins, respectively. This pattern is similar to that obtained with the WT construct (Figure 2A). The identity of the various truncated GST-α-Hbs was attested by Western blotting using anti-α globin antibodies (Figure 2B). The protein expression level of these GST-α-Hbs was similar to that of the WT construct except for the truncated GST-α-Hb1-117 protein for which a clearly decreased intensity was observed (Table 3). The expected decreased size of the proteins (Table 1) for the GST-α-Hb1-126, GST-α-Hb1-123 and GST-α-Hb1-117 mutant was observed (Figure 2B).

**Table 3:** Presence of different truncated α-Hbs observed by SDS-PAGE or by Western blot.

	SDS-PAGE				Western Blot			
truncated α chain	Expression	Solubilization		Purification	Expression	Solubilization		Purification
		soluble fraction	insoluble fraction			soluble fraction	insoluble fraction	
**α-Hb^WT^** *	**++**	**++**	**+**	**++**	**++**	**++**	**+**	**++**
**α-Hb1-138**	**++**	**++**	**+**	**++**	**++**	**++**	**+**	**++**
**α-Hb1-134**	**++**	**+**	**++**	**+**	**++**	**++**	**++**	**++**
**α-Hb1-126**	**++**	**−**	**++**	**−**	**++**	**+**	**++**	**+**
**α-Hb1-123**	**++**	**−**	**++**	**−**	**++**	**−**	**++**	**−**
**α-Hb1-117**	**+**	**−**	**++**	**−**	**+**	**+**	**++**	**+**

++: normal presence; +: decreased presence; **−**: absence or highly decreased presence.

*α-Hb^WT^ is recombinant wild type α-Hb1-141 with a N-terminal Gly-Pro-Leu-Gly-Ser peptide.

**Figure 3:**
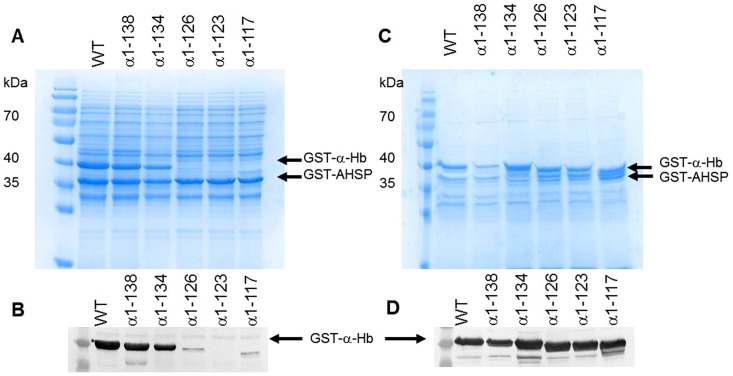
Effect of truncation on the solubility of various truncated α-Hbs. The induced *E.coli* BL21(DE3) cells containing different pGEX-α-AHSP plasmids were disrupted. After centrifugation, soluble fractions were analyzed by (A) SDS-PAGE (12%) and by (B) Western Blotting using anti-α globin antibodies. The insoluble fractions were analyzed by (C) SDS-PAGE (12%) and by (D) Western Blotting using anti-α globin antibodies. Page ruler™ prestained protein ladder (Fermentas Thermo Fisher Scientific) was in the first lane.

After solubilization, only the GST-α-Hb1-138 and GST-α-Hb1-134 were observed in the supernatant by SDS-PAGE analysis (Figure 3A) indicating their good solubility. These results were confirmed by Western blotting using anti-α globin antibodies (Figure 3B). To evaluate the amount of truncated GST-α-Hbs remaining in the pellet, the insoluble fraction was also analyzed by SDS-PAGE (Figure 3C). The GST-α-Hbs are clearly present but less abundant in the case of the truncated GST-α-Hb1-138, which was mostly present in the supernatant (Table 3). The same trend was observed by Western blotting analysis (Figure 3D), indicating that the truncated α-Hb1-126, α-Hb1-123 and α-Hb1-117 are expressed but are poorly soluble.

**Figure 4:**
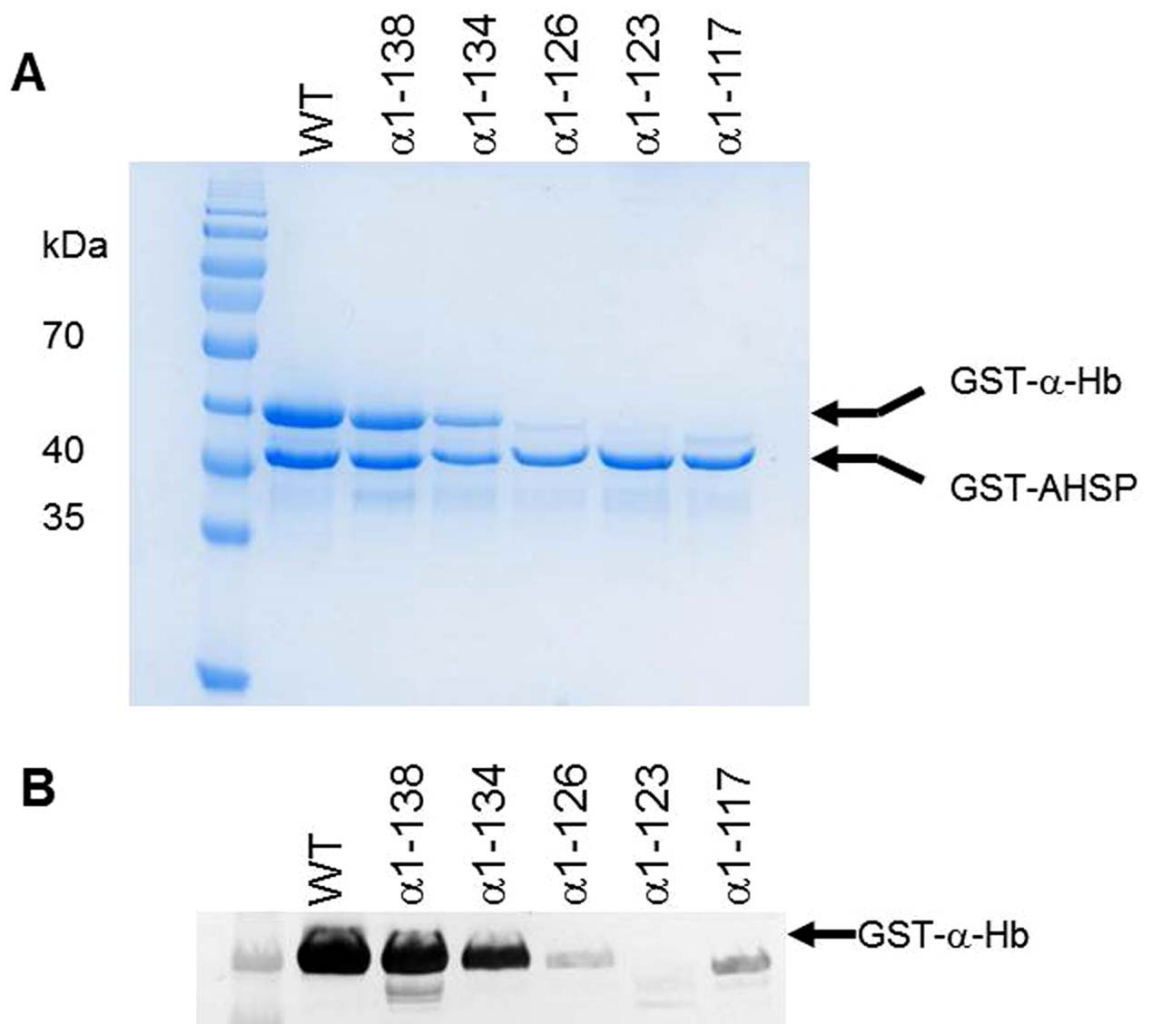
Yield of various truncated α-Hbs after purification. The different soluble fractions obtained after disruption of induced cells was applied on Glutathione Sepharose 4B in PBS (150 mM NaCl, 10 mM Na_2_HPO_4_, pH 7.4). After washing, various truncated GST-AHSP^WT^/GST-α-Hb complexes were eluted (elution buffer: 50 mM Tris-HCl, 20 mM reduced glutathione pH 8.0) and analyzed by (A) SDS-PAGE (12%) and (B) Western Blotting using anti-α globin antibodies.

Finally, SDS-PAGE analysis after purification of the different soluble fractions is shown in Figure 4A. Two major bands, 42 kDa and 38.6 kDa, were observed corresponding to the different GST-α-Hbs and GST-AHSP^WT^ proteins for the normal construct as well as for the truncated α-Hb1-138 and α-Hb1-134. In contrast, for the truncated α-Hb1-126, α-Hb1-123 and α-Hb1-117, no band corresponding to GST-α-Hb was visible. However, the Western blot analysis using anti-α antibodies revealed the presence of a band corresponding to GST-α-Hb (Figure 4B). Nevertheless, the intensity of the bands for α-Hb1-126 and α-Hb1-117 were drastically decreased and that of α-Hb1-123 was at the limit of detection. Thus, while the truncated α-Hb1-138 and α-Hb1-134 were purified in quantity, the truncated α-Hb1-126 and α-Hb1-117 were obtained in very low quantities and for the truncated α-Hb1-123 no further study was possible.

### Spectroscopic characteristics of the complexes

**Figure 5:**
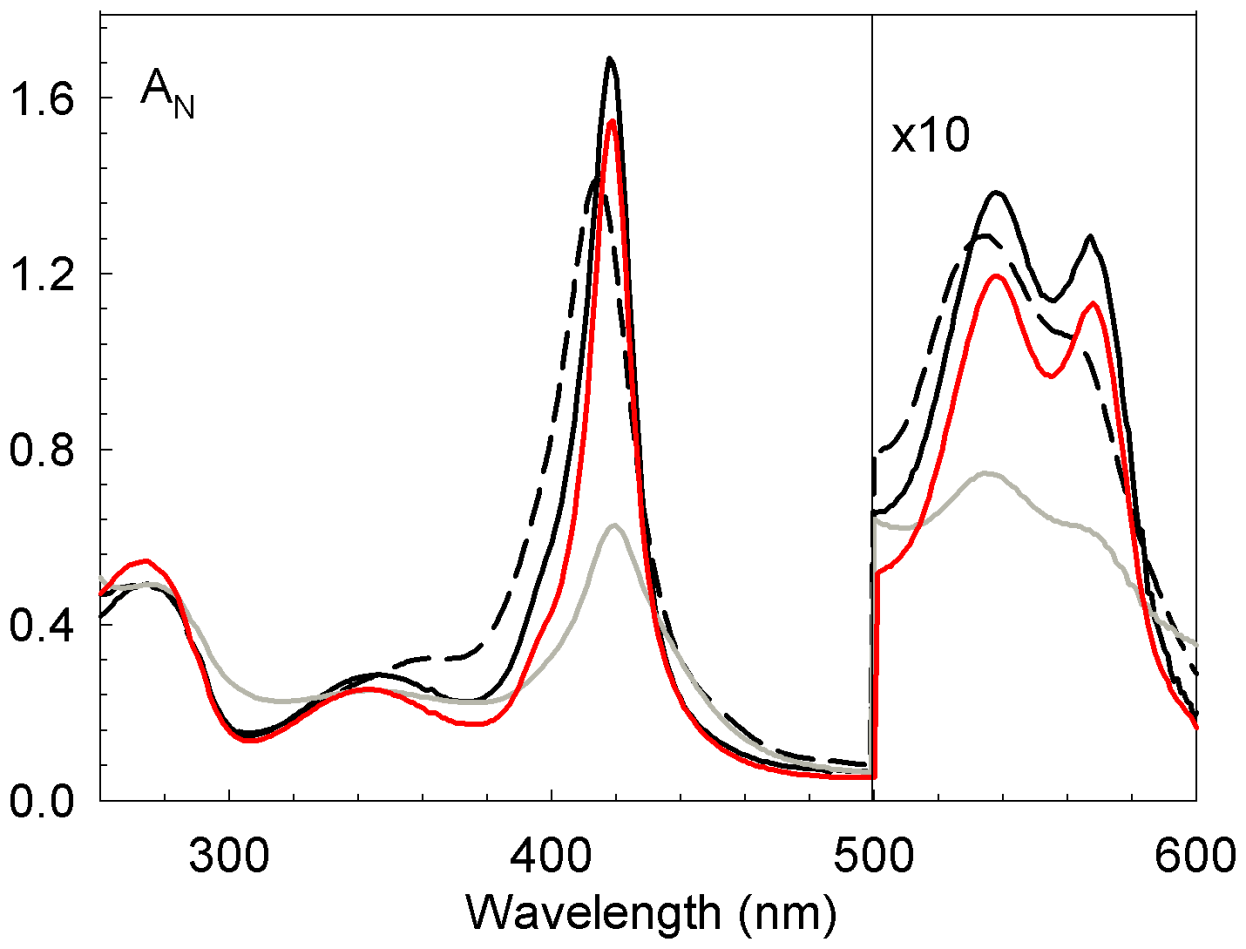
Absorption spectra of truncated AHSP^WT^/α-Hbs complexes. The spectra are represented in black for AHSP^WT^/α-Hb^WT^ complex, in red for of AHSP/α-Hb1-138 complex, in black dashed line for AHSP/α-Hb1-134 complex and in grey for AHSP/α-Hb 1-126 complex. The measurements were performed in PBS pH 7.4 at 25°C.

Native Hb A and free α-Hb exhibit characteristic spectra attesting the presence of the heme molecule with Soret γ band (415–420 nm), α and β absorbance peaks (540 and 571 nm) and δ-band (344 nm) [20]. The spectra of the AHSP^WT^/truncated α-Hb complexes are shown in Figure 5. The relative contributions at 280 nm, a wavelength which is sensitive to the aromatic amino acids and heme, and at visible wavelengths which are essentially due to the heme contribution can provide information on the heminization state of truncated α-globin. The spectra of the AHSP^WT^/α-Hb^WT^ (solid black line in Figure 5) and AHSP^WT^/native α-Hb complexes are similar [21]. The spectrum observed for AHSP^WT^/α-Hb1-138 (solid red line in Figure 5) is similar to that observed for the AHSP^WT^/α-Hb^WT^ complex, while the spectrum of the AHSP^WT^/α-Hb1-134 complex exhibits a decreased amplitude of the Soret band and an asymmetry of the α and β bands (black dashed line in Figure 5). These differences are more marked for AHSP^WT^/α-Hb1-126 complex with a Soret band decreased by a factor 2.7 and an asymmetry of the α and β bands (solid grey line in Figure 5). In the case of the AHSP^WT^/α-Hb1-117 complex, an abnormal UV-visible spectrum is observed with an absence of characteristic bands of the heme molecule showing that most of globin has not incorporated the heme molecule. These results indicate that the more H helix is truncated, the less heme molecule incorporates into the α-globin (Table 4).

**Table 4:** Characteristics of different truncated AHSP^WT^/α-Hb complexes.

	Spectroscopy		CO binding kinetics		CD Spectroscopy		Quenching of Fluorescence**	
	UV	Visible		+ β-Hb*	α -helix (%)	Tm		+ β-Hb
Controls								
AHSP^WT^	++	−	−	−	60	55.6–58	no	no
native αHb AHSP^WT^/native α-Hb AHSP^WT^/α-Hb^WT^	++ ++ ++	+++ +++ +++	100% R 100% I 100% I	R and T state R and T state 56% R, 32% T	71 67 65	64 63 60	++++ +++ +++	++++ + +
AHSP^WT^/α-Hb1-138	++	+++	97% R	95% R, 5% I	74	63	+++	+
AHSP^WT^/α-Hb1-134	++	++	10% I, 87% R	84% R% 12% I	71	56	++	+
AHSP^WT^/α-Hb1-126	++	+	19% I, 81% R	80% R, 20% I	nd	nd	++	++
AHSP^WT^/α-Hb1-117	+++	no	20% I, 80% R	88% R, 12% I	nd	nd	nd	nd

*The CO recombination kinetics were measured in the presence of IHP.

**The fluorescence signal of globin and AHSP^WT^/α-Hb^WT^ complex is highly quenched by the heme molecule demonstrating the interaction between AHSP^WT^ and α-Hb^WT^.

### Effects of truncation on secondary structure and thermal stability of AHSP^WT^/α-Hb1-138 and AHSP^WT^/α-Hb1-134

To assess the consequences of end extremity truncation on the structure and stability of α-Hb chain, we have first analyzed by CD spectroscopy the AHSP^WT^/α-Hb1-138 and AHSP^WT^/α-Hb1-134 complexes in the CO form. The far UV CD revealed that the AHSP^WT^/α-Hb1-138 and AHSP^WT^/α-Hb1-134 complexes display an α-helical content of 74% and 71%, respectively. These α-helical contents are slightly higher than for AHSP^WT^/native α-Hb (67%) or AHSP^WT^/α-Hb^WT^ (65%) (Table 4) and can be explained by the absence of three C-terminal residues following the helical structure.

**Figure 6:**
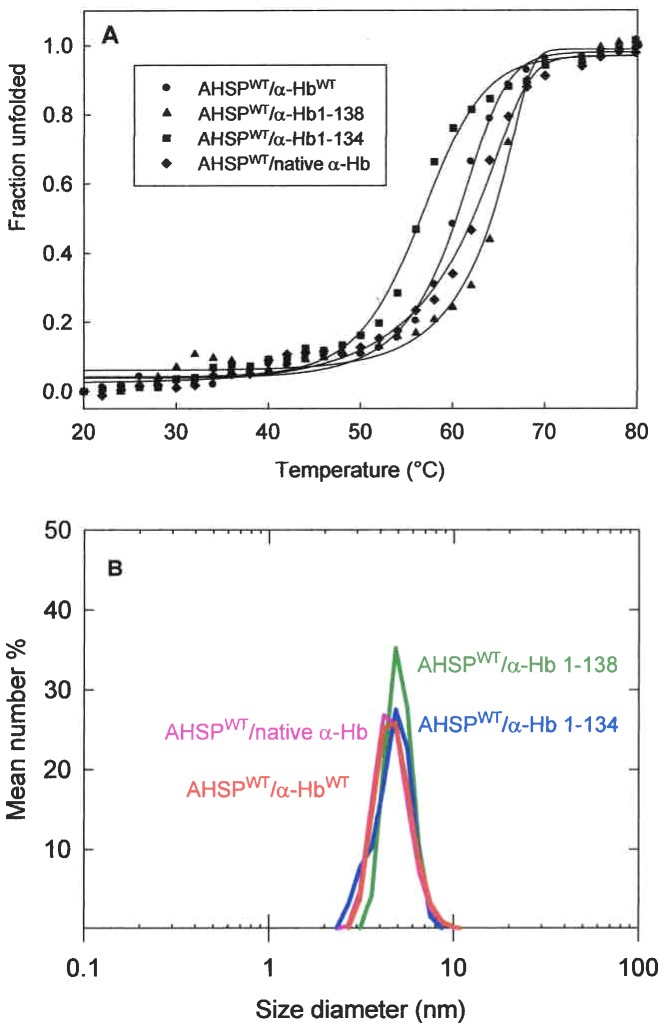
Conformational Stability of truncated AHSP^WT^/α-Hb complexes. (A) Thermal unfolding curve measured by CD experiments. Fraction unfolded (fu) is versus temperature for AHSP^WT^/native α-Hb (♦), AHSP^WT^/α-Hb^WT^ (•), AHSP^WT^/α-Hb1-138, (▴) and AHSP^WT^/α-Hb1-134 (▪) complexes. The solid lines are a smooth curve (sigmoidal representation) to help estimate Tm. Protein concentration was around 20 µM (on a heme basis) in 2.5 mM Na_2_HPO_4_, 37.5 mM NaCl buffer at pH 7.4 in the presence of sodium dithionite at 1 mM. The change in ellipticity was recorded at 222.6 nm from 20 to 80°C with a heat rate of 1°C.min^−1^. (B) Hydrodynamic diameter for the complexes of AHSP^WT^ with native α-Hb, α-Hb^WT^, α-Hb1-138 and α-Hb1-134. Protein concentration was around 20 µM (on a heme basis) in PBS.

The instability of the protein was measured by thermal denaturation using the CD and by determination of the melting temperature (Tm) for which 50% of molecules are unfolded. The AHSP^WT^/α-Hb^WT^ and AHSP^WT^/native α-Hb complexes exhibit a Tm of 60°C and 63°C, respectively (Figure 6A). For AHSP^WT^/α-Hb1-138 complex, the Tm (63°C) is close to that observed for WT recombinant complex or for isolated native α-Hb (64°C). In contrast, a decreased Tm value for AHSP^WT^/α-Hb1-134 complex (56°C) was found (Table 4).

Secondly, we investigated the effect of truncation on the hydrodynamic diameter of complexes based on DLS. In the same experimental conditions, AHSP^WT^, small protein consisting of an elongated α-helix bundle [5] exhibits a hydrodynamic diameter about 3.5 nm, while that for the more globular tetrameric Hb is about 6 (data not shown). It is important to note that AHSP^WT^/native α-Hb and AHSP^WT^/α-Hb^WT^ complexes exhibit a similar size of hydrodynamic diameter, 4.3 and 4.5 nm respectively (Figure 6B) although there are 5 additional amino acids (Gly-Pro-Leu-Gly-Ser) for each recombinant protein. For AHSP^WT^/α-Hb1-138 and AHSP^WT^/α-Hb1-134 complexes, one observes a slight increase of the size of hydrodynamic diameter indicating that the truncation of the C-terminal end does not modify the size of these complexes compared to those observed for WT complex.

### Truncation effects on fluorescence of various complexes

**Figure 7:**
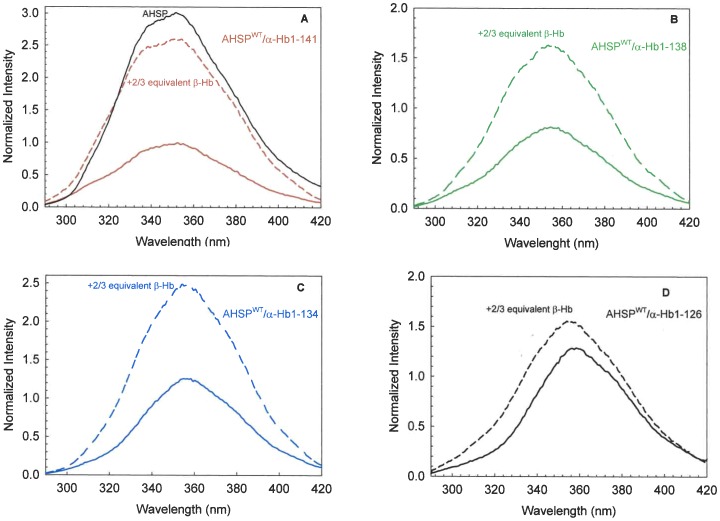
Fluorescence emission spectra of AHSP^WT^/α-Hb complexes before and after addition of β-Hb. (A). Fluorescence emission spectra of AHSP^WT^/α-Hb^WT^ complex; (B). Fluorescence emission spectra of AHSP^WT^/α-Hb1-138 complex; (C). Fluorescence emission spectra of AHSP^WT^/α-Hb1-134 complex; (D). Fluorescence emission spectra of AHSP^WT^/α-Hb1-126 complex. The fluorescence emission spectrum of AHSP^WT^ is shown in the figure 7A (solid black line). The concentrations are around 3 µM (on a heme basis) in PBS. Each different emission spectrum of AHSP^WT^/truncated α-Hb complex is normalized with respect to the emission maxima obtained for normal complex. The dashed lines illustrate the fluorescence emission spectra of different AHSP^WT^/α-Hb complexes after addition of β-Hb chains (2/3 equivalent).

The fluorescence energy transfer technique was used to investigate the interaction between AHSP and truncated α-Hb within the AHSP^WT^/α-Hb complexes. AHSP has a single Trp (position 44) and exhibits a fluorescence spectrum typical of an exposed Trp (solide black line in Figure 7A). α-Hb has also a single Trp at position 14 (A2 in the A helix) but the α-Hb fluorescence is highly quenched by its heme group. It is important to note that there is no difference in fluorescence emission spectra between AHSP^WT^/native α-Hb and AHSP^WT^/α-Hb^WT^ complexes as illustrated in the Figure S1. In Figure 7 are illustrated the different emission spectra obtained for different complexes after normalization with respect to the emission maxima obtained with the WT complex. The emission spectrum of AHSP^WT^/α-Hb^WT^ complex is highly decreased compared to AHSP^WT^ (solid red line and solid black line respectively, in Figure 7A), the quenching of AHSP fluorescence intensity demonstrating the interaction between AHSP and α-Hb [21]. Only the AHSP^WT^/α-Hb1-138 complex exhibits an emission spectrum with a slightly decreased fluorescence signal compared to that AHSP^WT^/α-Hb^WT^ complex (solid line in Figure 7B). The AHSP^WT^/α-Hb1-134 and AHSP^WT^/α-Hb1-126 complexes (solid lines in Figure 7C and 7D) have emission spectra with an increased amplitude, due to less heme molecule and therefore less quenching. These data are in agreement with absorbance spectra results (Figure 5). For all truncated complexes, one observes a red shift of fluorescence indicating that the environment of Trp is more polar.

Addition of β-Hb subunits to the AHSP^WT^/α-Hb^WT^ complex (dashed line in Figure 7A) leads to an increase in fluorescence intensity demonstrating the release of the part of AHSP^WT^ and the formation of dimeric (αβ) Hb [21]. Addition of β-Hb to the AHSP^WT^/truncated α-Hb complexes leads to an increase in fluorescence intensity in all cases, as observed for WT complex (dashed lines in Figure 7 B,C,D). These results indicate that all truncated α-Hb into the AHSP^WT^/α-Hb complexes are able to release AHSP for replacement by the β-Hb chains.

### Effect of truncation on CO binding kinetics of various complexes

**Figure 8:**
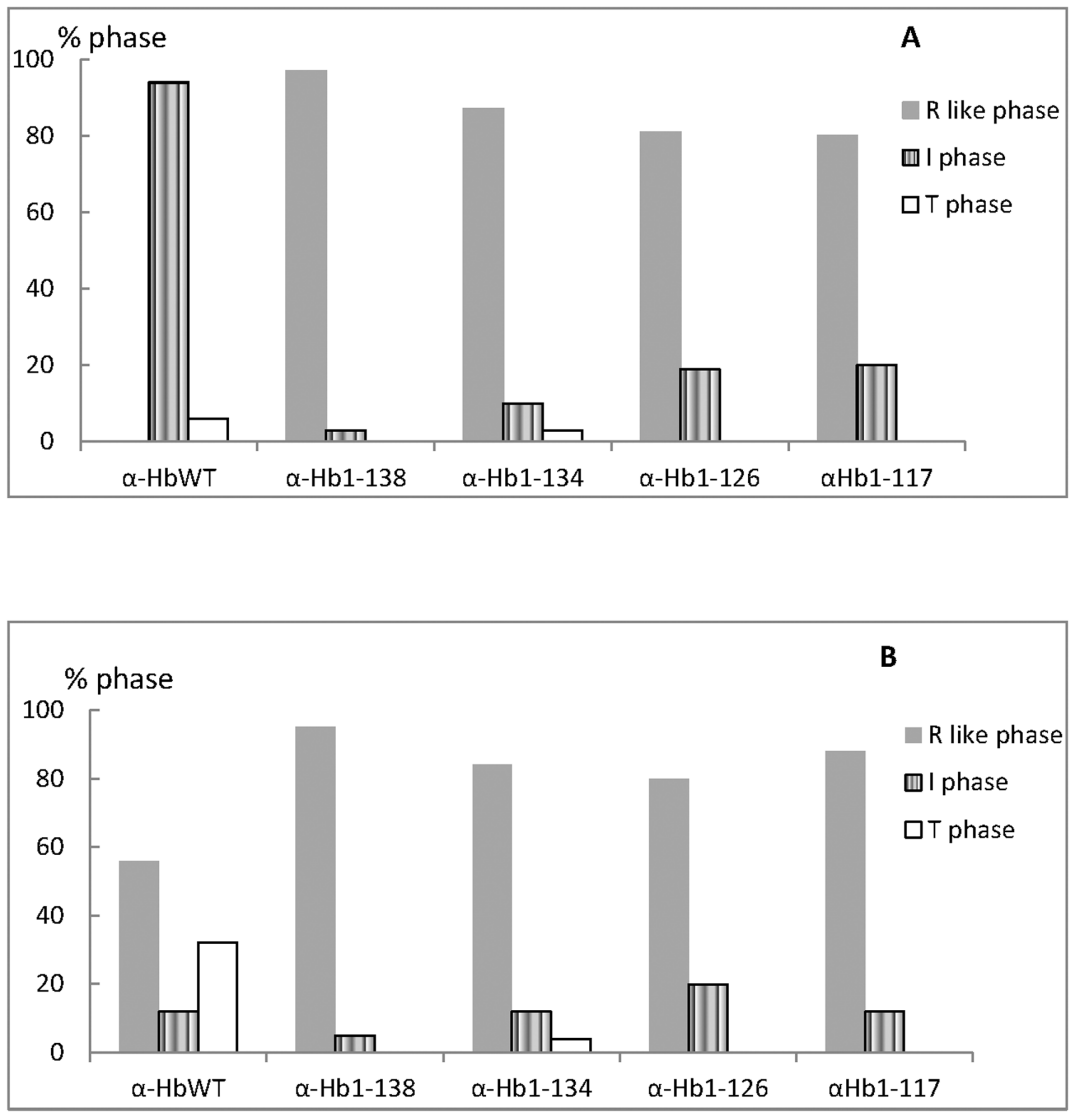
Ligand binding studies of the various truncated GST-AHSP^WT^/GST-α-Hb complexes (A) and after addition of β-Hb in the presence of IHP (B). The CO recombination kinetics reveal whether the protein complexes display the known rates for the classical R and T states, or the reference intermediate rate for the AHSP^WT^/α-Hb^WT^ complex. The truncated forms of α-Hbs complexed with AHSP show less of the I state, indicating a modified interaction. Usually, the addition of β-Hb in the presence of IHP provokes a replacement of the AHSP by the β-Hb and leads to formation of Hb dimers and tetramers which display the usual slow T-state kinetics; however, the truncated forms displayed little of this allosteric form. Experimental conditions were 50 mM Bis-Tris buffer at pH 7.0, 100 mM CO at 25°C in absence or in presence of IHP at a final concentration of 1 mM. The concentration of different complexes on heme basis varies between 3.4 and 10.6 µM depending on the truncation.

Tetrameric Hb presents CO binding kinetics including a rapid phase (CO association rate of 6 µM^−1^s^−1^) and a slow phase (CO association rate of 0.2 µM^−1^s^−1^) corresponding to the R and T states, respectively. Unlike isolated α- and β-Hb subunits, which display CO binding kinetics typical of the R-state, α-Hb in the presence of AHSP exhibits a rate of about 2 µM^−1^s^−1^, intermediate to the R and T states of tetrameric Hb, and was thus denoted as the I phase [21]. We measured the influence of α-Hb chain truncation on CO recombination kinetic of different complexes (Figure 8A). These measurements were made directly on GST-AHSP^WT^/GST-α-Hb complexes because the GST moiety does not interfere with the CO recombination kinetics [19], [21]. Unlike AHSP^WT^/α-Hb^WT^ complex, the CO binding kinetic of different truncated complexes did not show mainly the intermediate phase but a rapid phase typical of R-state with a CO association rate of 6.5 µM^−1^s^−1^. The α-Hb1-138 complex exhibits nearly 100% of the rapid phase. The other truncated α-Hb complexes display between 10% and 20% of the I phase (Figure 8A and Table 4).

After addition of native β-Hb and inositol hexaphosphate (IHP), an allosteric effector stronger than 2,3 diphosphoglycerate that increases the fraction of slow (T-state) phase, the kinetics for the WT complex become biphasic demonstrating the formation of functional Hb tetramers [21] with both R and T phases, corresponding to the two allosteric states. In contrast, for truncated α-Hb complexes, mainly the R phase was observed indicating the loss of allostery (Figure 8 B, Table 4).

## Discussion

It is now admitted that normally α-Hb first binds AHSP, this chaperone maintaining the α-Hb in a soluble state until association with the β-Hb chain partner to form α_2_β_2_ tetramer [1], [2], [3]. Different abnormal α-Hbs are described as being unstable because leading to a low abundance of the abnormal Hbs in the RBCs [18]. The amino acid changes, deletions or insertions of some of these unstable Hbs are located in the G or H helices which are involved in the interaction with protein partners, AHSP or β-Hb. Different molecular mechanisms have been proposed to explain the instability of these mutated α-chains [22]. In the case of the mutants with elongated chains (Table 2), it has been proposed that the mutated α-globin mRNA is unstable with a shortened life span contrasting with the normal globin mRNAs considered as being highly stable [23]. Another possibility is that the abnormal α chains are unable to properly bind AHSP by disruption of hydrophobic interactions between helices.

In this study, we investigated the interaction of the H helix of α-Hb with AHSP using as probe different natural α-Hb variants known as being unstable in the RBCs (Table 2). Instability of native α-Hb is well known [24] and in a previous study, we have shown that it is possible to express normal α-Hb alone as GST fusion protein, but it is not recovered after the solubilization step despite the presence of the GST moiety that can help to stabilize the folding of recombinant proteins [19]. So the presence of the protein partner is required to produce soluble recombinant α-Hb. Its co-expression with AHSP permits one to obtain the GST-α-Hb associated with AHSP in the heterodimer form (GST-AHSP^WT^/GST-α-Hb) [19]. Thus the five α-Hb variants with shortened H helix were engineered in association with AHSP.

The SDS-PAGE results showed that the truncation of the α-globin chain did not alter the expression of the GST-α-Hb protein except in the case of GST-α-Hb1-117 where it was decreased (Figure 2). This normal expression of different GST-α-globins suggests that their mRNAs are stable in BL21 (DE3) cells. An instability of mRNA for GST-α-Hb1-117 could be considered, but a further study would be required to confirm such a hypothesis.

After the step of solubilization, the truncated α-globin chains exhibit variable solubilities. When the globin has a length of 134 amino acids residue, the protein is mainly in the soluble form. Conversely when shortened to a length of 126 amino acids α-globin it can be does not permit to maintain soluble the truncated α-globin chains. Thus, SDS-PAGE and Western blotting analysis showed that when the H helix was truncated, less complex was formed. This reveals an impaired attachment or interaction between truncated α-Hb and AHSP. In the case of GST-α-Hb1-123, the absence of this protein after solubilization may be partially explained by the absence of residue 129 (H17) which interacts with AHSP (Figure 1A). Indeed we showed in a previous study that the substitution of Leu129 by Pro residue perturbs the interaction with AHSP and explains the absence of abnormal Hb in the RBCs and the α^+^-phenotype of the patient [16].

Another parameter that can enhance the stability, and facilitate the correct folding of the α apoglobin, is the presence of exogenous heme during expression. Indeed it has been demonstrated that the co-expression of bacterial membrane heme transport increase the rate and extent of the heme capture through the bacterial membranes [25]. It has been also shown in a wheat germ cell-free translation system that nascent α-globin having 86 amino acids can interact with hemin [26]. Consequently, in our expression conditions where hemin molecule is added in the same time than the inductor of globin synthesis, it is reasonable to assume that the truncated α-globins have enough amino acids to bind heme. Indeed the absorbance spectra reveal well the presence of heme for truncated α-Hb1-138, α-Hb1-134 and α-Hb1-126 complexes (Figure 5). While the AHSP^WT^/α-Hb1-138 exhibits a similar spectrum than this of WT complex, the AHSP^WT^/α-Hb1-134 exhibits some spectral modification and the AHSP^WT^/α-Hb1-126 complex presents a highly decreased amplitude of the Soret band indicating a low heminization. The spectrum of the truncated α-Hb1-117 does not exhibit the characteristic bands of the heme molecule (data not shown). Three amino acid residues in the H helix are in contact with the heme; the 129(H12) residue at the distal region and 132(H15) and 136(H19) residues at the proximal region (Figure 1A). In the truncated α-Hb1-126 and α-Hb1-123 where these three residues are deleted, the different results reflect the decreased interaction with heme. In addition for the two truncated α-Hbs, there is the loss of one contact (position 129 (H17)) with AHSP can explain the decreased quantity of purified proteins. However these different results do not explain why the shorter truncated α-Hb1-117 is obtained in sufficient quantity for some studies while for the truncated α-Hb1-123, we do not obtain this α-Hb after purification. One could hypothesize that in this last truncated α-Hb, the presence of only 6 residues of H helix including a proline residue is more destabilizing than the total absence of the H helix.

Study of the fluorescence signal is a good method to evidence modifications in the interactions between the different truncated α-Hbs, AHSP and heme. In a previous study, we have shown that quenching of the fluorescence signal of the single Trp residue of AHSP may be used as a probe for its binding to α-Hb chain. The heme group being an excellent energy acceptor, strongly quenches the globin fluorescence as well as that of interacting partner proteins [21]. The fluorescence studies clearly show an increase in fluorescence intensity emission for the AHSP^WT^/α-Hb1-134 and AHSP^WT^/α-Hb1-126 complexes compared to that observed for the WT complex (Figure 7C,D) and indicate that a fraction of the truncated α-Hb is not associated to the heme group and is present in α apo-globin form. The studies of fluorescence properties of α-globin have shown that its fluorescence emission spectrum exhibited emission maxima at 335±2 nm which is characteristic of an apolar environment [27]. This data is in agreement with crystallographic studies which showed that the Trp 14(A12) of the α-chain is an internal residue [28], [29]. For AHSP^WT^/α-Hb1-134 and AHSP^WT^/α-Hb1-126 complexes, a red shift is observed that may be due to the presence of apo α-globin but with a different structure of that observed for WT α-globin in particular the Trp 14(A12) will be in the more polar environment and this may be related to intramolecular structural rearrangements. These modifications of the structure in AHSP^WT^/α-Hb1-134 can explain the diminution of stability found by CD results. In addition, the loss of heme molecule may also have consequences in the fluorescence emission of the AHSP Trp. Increase of the fluorescence signal upon addition of β-Hb to the truncated α-Hbs demonstrates the release of AHSP and the further formation of dimers and tetramers. Finally results of the CO binding kinetics show that the truncated AHSP^WT^/α-Hb complexes do not exhibit the intermediate phase as observed for the AHSP^WT^/α-Hb^WT^ (Figure 8A). The CO binding kinetics of the Hb tetramers incorporating the truncated α-Hbs revealed that these molecules were functionally abnormal (Figure 8B).

In summary, the deletion of the C-terminal extremity including the last three amino acids has no effect on the stability of the molecule. The end of helix H (134–138) is a region that does not interact with either AHSP or β-Hb subunits, so its deletion does not greatly influence the stability of α-globin. In contrast, the region 129-137 is involved in the interaction with heme; in particular the absence of the three amino acids (129, 132 and 136) as observed in the truncated α-Hb1-126, α-Hb1-123 and α-Hb1-117 leads to a decrease of heme incorporation and abnormal function of these α-globin chains. Finally the N-terminal extremity of the H helix is primordial to the interaction with AHSP. The data presented here clearly show that the stability of α-globin chain requires the interaction between AHSP and heme molecule at different sites of the H helix.

## Materials and Methods

### Site-directed mutagenesis

The different stop codons were introduced at the *ad hoc* position of α-globin by site-directed mutagenesis (Quick change Lightning Site Directed Mutagenesis kit, Stratagene, Agilent technologies, Santa Clara, CA, USA) using the pGEX6P-α-AHSP vector as template [19]. Primers were purchased from Eurofins MWG Operon (Ebersberg, Germany) and are described in Table 5. Once the different stop codons were introduced, the integrity of the human α-globin and AHSP cDNA coding regions were checked by DNA sequencing (Eurofins MWG Operon).

**Table 5:** Primer sequences for site directed-mutagenesis.

Name of truncated α chain	primer sequences (5′-3′)
α-Hb1-138 Forward	CCGTGCTGACCTCCTAATACCGTTAACTC
α-Hb1-138 Reverse	GAGTTAACGGTATTAGGAGGTCAGCACGG
α-Hb1-134 Forward	GGCTTCTGTGAGCACCTAGCTGACCTCCAAATACC
α-Hb1-134 Reverse	GGTATTTGGAGGTCAGCTAGGTGCTCACAGAAGCC
α-Hb1-126 Forward	GCACGCCTCCCTGGACTAATTCCTGGCTTCTGTGA
α-Hb1-126 Reverse	CTCACAGAAGCCAGGAATTAGTCCAGGGAGGCGT
α-Hb1-123 Forward	CTGCGGTGCACGCCTAACTGGACAAGTTCCTG
α-Hb1-123 Reverse	CAGGAACTTGTCCAGTTAGGCGTGCACCGCAG
α-Hb1-117 Forward	CTCCCCGCCGAGTTCTAACCTGCGGTGCACGCC
α-Hb1-117 Reverse	GGCGTGCACCGCAGGTTAGAACTCGGCGGGGAG

Couples primers containing the mutations are designed *in silico* using the PrimerX software (http://www.bioinformatics.org/primerx/) following the manufacturer's recommendations. The primers were synthesized *in vitro* and purified by HPSF (High Purity Salt Free or Highly purified salt-free, Eurofins MWG Operon).

### Expression and purification of AHSP^WT^/α-Hb complexes

WT and truncated recombinant α-Hbs were co-expressed with AHSP^WT^ in *E.coli* BL21(DE3) cells (Lucigen, Middleton, USA) as two fusion proteins with GST, GST-α-Hb and GST-AHSP [19]. Briefly, after induction by 0.2 mM isopropyl β-thiogalactipyranoside (IPTG) at 37°C and supplementation with hemin (30 µg/mL), the growth was continued for 4 hours. Bacteria were then harvested by centrifugation. Pellets were dissolved in Phosphate Buffered Saline (PBS) (150 mM NaCl, 10 mM Na_2_HPO_4_, pH 7.4) in the ratio of 1 mL PBS for 5 mL of culture and then frozen at −80°C. After thawing, 5 mM dithiothreitol (DTT) and 1 g lysozyme/Liter were added to the resuspended pellets and incubated on ice for 30-min. The lysis was completed by brief ultrasonic pulses using a Sonifier II disrupter (Branson Ultrasonic, Carouge-Geneva, Switzerland). The obtained solution was incubated in the presence of 1% Triton X-100 for 1 h at 4°C. The homogenate was then centrifuged at 14 000 rpm for 30 min at 4°C. The different supernatants containing soluble GST-AHSP^WT^/GST-α-Hb complexes were recovered, gazed with CO and purified by a single step of affinity chromatography on Glutathione Sepharose 4B (GE Healthcare Lifesciences, Uppsala, Sweden) [19]. The insoluble fraction remaining after solubilization was resuspended in one volume of PBS containing 10% sodium dodecyl sulfate (SDS), sonicated and analyzed by SDS-PAGE and western blotting.

For AHSP^WT^/α-Hb complex studies, direct protease cleavage of GST proteins bound to glutathione beads was achieved by the addition of the Prescission Protease (80 U/mL glutathione beads) (GE Healthcare Lifesciences) in PBS containing 1 mM DTT overnight at 4°C under gentle agitation. The released recombinant proteins were recovered in the supernatant after centrifugation, while GST moiety and Prescission Protease remained bound to the matrix. The recombinant proteins were concentrated by ultracentrifugation (Amicon Ultra 10 kDa) and gazed with CO. Then AHSP^WT^/α-Hb complexes were purified in PBS, on Superose^TM^12 HR 10/300 GL column (GE Healthcare Lifesciences) adapted to Akta Purifier 10 (GE Healthcare Lifesciences) FPLC-Fast Protein liquid chromatographic system.

### Preparation of AHSP^WT^/native α-Hb complex

The GST-AHSP^WT^/native α-Hb complex was prepared as previously described [21]. Briefly, after reaction of human Hb with p-hydroxymercuribenzoic acid, the isolated α-Hb chains were purified by ion exchange chromatography, then saturated with CO and stored at -80°C. AHSP^WT^ was expressed as a GST fusion protein and purified as described above. The GST-AHSP^WT^/native α-Hb complex was obtained after adding an excess of carbonmonoxy α-Hb chains to GST-AHSP^WT^ fixed to glutathione-Sepharose 4B beads (GE Healthcare Lifesciences).

### Gel electrophoresis and western blotting

Ten microliters of aliquots at each step of the recombinant protein production were analyzed by SDS-PAGE as described by Laemmli [30] using 12% polyacrylamide gels (acrylamide:bisacrylamide 37.5∶1). Protein bands were stained by Coomassie Brilliant Blue R, scanned with a GeldocXR^+^ system (Bio-Rad Life Science, Hercules, CA USA) and analyzed by Image Lab™ software version 3.0 (Bio-Rad Life Science). After SDS-PAGE, the proteins were transferred to hydrophobic polyvinylidene difluoride (PVDF) membrane (Hybond-P, GE Healthcare, Life Sciences). Membranes were rinsed briefly in Tris-buffered saline (10 mM Tris HCl pH 7.5, 150 mM NaCl) containing 0.05%Tween 20 (TBST). PVDF membranes were blocked with TBST containing 2% bovine serum albumin for 3 h and then incubated at 4°C over-night with primary monoclonal mouse anti-α-Hb antibodies (Abnova, Taipei, Taiwan) diluted 1∶2000 in TBST.

After primary antibody incubations, PVDF membranes were washed 3 times with TBST and incubated with secondary antibody conjugated with alkaline phosphatase at a dilution of 1∶1500 for 2 h at room temperature. Binding proteins were visualized using a tablet of 5-Bromo-4-Chloro-3′-Indolyphosphate p-Toluidine/Nitro-Blue Tetrazolium (SIGMAFAST BCIP/NBT, Sigma-Aldrich, Saint Louis, MO, USA) following the manufacturer's instructions.

All experiments were repeated at least three times.

### UV-visible spectroscopic measurements

UV-visible spectra of different truncated GST-AHSP^WT^/GST-α-Hb or AHSP^WT^/α-Hb complexes were carried out with an Hewlett-Packard 8453 spectrophotometer. Spectra of complexes in CO form were recorded between 200 to 700 nm at room temperature using 1 cm path-length quartz cuvettes. All spectra were duplicated on two different preparations.

### CD measurements and thermal denaturation

CD spectra were collected from 190 to 260 nm on a Jasco spectropolarimeter (model J-810) (Tokyo, Japan) equipped with a thermo-stated cell holder at 20°C under a constant nitrogen flow. Cuvettes with 0.5 mm path length were employed. Each spectrum was the average of five scans, each normalized against the 2.5 mM Na_2_HPO_4_, 37.5 mM NaCl buffer at pH 7.4. Far-UV CD spectra were collected with a step resolution of 0.1 nm, a scan speed of 100 nm per minute and a bandwidth of 2 nm. The CD spectra were deconvoluted using CDNN software (Bohm, Halle, Germany, http://bioinformatik.biochemtech.uni-halle.de/cdnn). The different AHSP^WT^/α-Hb complexes were equilibrated under 100% CO at a concentration of 18–20 µM (on a heme basis) in 2.5 mM Na_2_HPO_4_, 37.5 mM NaCl buffer at pH 7.4 in the presence of sodium dithionite at 1 mM. To determine thermal denaturation curves, the ellipticity at 222.6 nm was monitored over a temperature range of 20–80°C, using a bandwidth of 1 nm, and a temperature gradient of 1°C per min. The CD signal was normalized to obtain the unfolded fraction: fu  =  (yN–yobs) / (yN–yu), where yobs is the observed CD signal and yN and yu the CD signal of the native and unfolded protein, respectively.

### DLS

Size distribution of different AHSP^WT^/α-Hb complexes was determined by DLS in a Zetasizer Nano-ZS (Malvern Instruments Ltd, Worcestershire, UK) with determination of the hydrodynamic diameter from the standard Stokes Einstein equation for spherical particle. Measurements were performed in PBS at 25°C and determined from the average of at least five measurements.

### Fluorescence measurements

All the fluorescence measurements were made on a Perkin Elmer fluorescence spectrophotometer (LS-55) (Waltham, MA, USA) with a 4×10 mm path length quartz cuvette. Both the excitation and emission band passes were kept at 2.5 nm and 5 nm, respectively. In all experiments, the fluorescence was followed using excitation at 280 nm and measuring the emission fluorescence spectra between 290 and 420 nm. The AHSP^WT^/α-Hb complexes were prepared in 100% CO and at a concentration of 3 µM (on a heme basis) in PBS containing 10 mM DTT and 1 mM sodium dithionite. The β-Hb equilibrated with 100% CO was added and a 10 minute incubation was made before the measurement.

### CO recombination kinetics after flash photolysis

The kinetics of CO recombination after flash photolysis on the different GST-AHSP^WT^/GST-α-Hb complexes were measured using 10 ns YAG laser pulses (Quantel, Les Ulis, France) at 532 nm [31], [32]. Samples at 5 µM on a heme basis, were analyzed in 3×3 mm quartz cuvettes with observation at 436 nm. Measurements were done at 25°C in 50 mM Bis-Tris at pH 7.0. Samples were equilibrated under 10% CO corresponding to about 100 µM CO. The different complexes were first studied alone. In the second series of experiments, β-Hb was added in the presence of 1 mM IHP.

## Supporting Information

Figure S1 **Fluorescence emission spectra of AHSP^WT^/α-Hb^WT^ complex compared to those obtained for AHSP^WT^/native α-Hb complex before and after addition different quantities of β-Hb.** The fluorescence emission spectrum of AHSP is shown in the solid black line. The concentrations are around 3 µM (on a heme basis) in PBS. The solid lines and dashed lines illustrate the fluorescence emission spectra of different AHSP^WT^/native α-Hb and AHSP^WT^/α-Hb^WT^ complexes, respectively.
(TIF)
