# Supplementary figures and images for: Role of α-Globin H Helix in the Building of Tetrameric Human Hemoglobin: Interaction with α-Hemoglobin Stabilizing Protein (AHSP) and Heme Molecule
Source: PLoS One. 2014 Nov 4;9(11):e111395. doi: 10.1371/journal.pone.0111395 (PMC4219717; doi:10.1371/journal.pone.0111395)

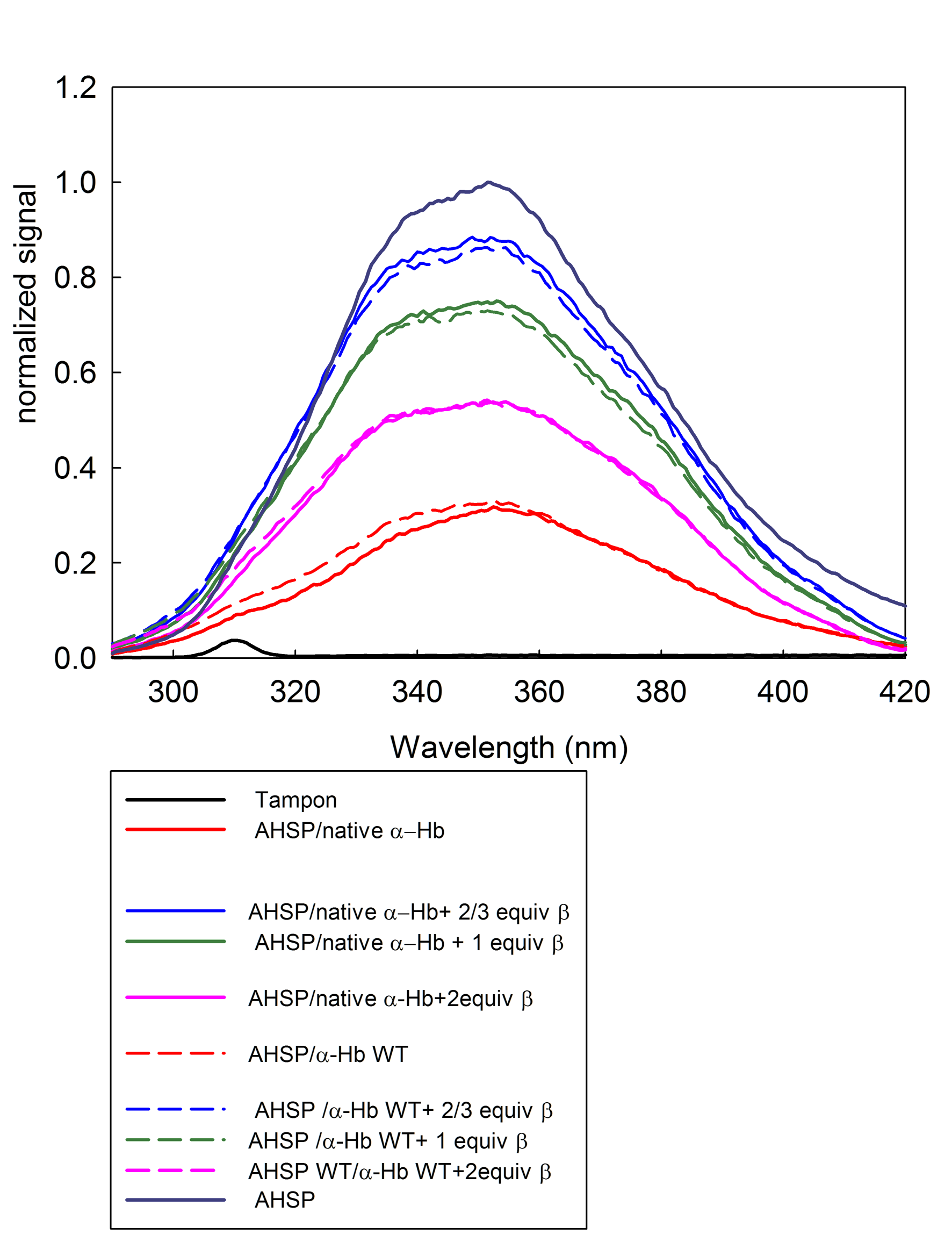

Supplement: Figure S1 — Fluorescence emission spectra of AHSPWT/α-HbWT complex compared to those obtained for AHSPWT/native α-Hb complex before and after addition different quantities of β-Hb. The fluorescence emission spectrum of AHSP is shown in the solid black line. The concentrations are around 3 µM (on a heme basis) in PBS. The solid lines and dashed lines illustrate the fluorescence emission spectra of different AHSPWT/native α-Hb and AHSPWT/α-HbWT complexes, respectively. (TIF) [file pone.0111395.s001.tif]
